# 发酵虫草菌粉及产品指纹图谱建立及多指标成分分析

**DOI:** 10.3724/SP.J.1123.2021.06022

**Published:** 2021-09-08

**Authors:** Wen CAO, Liang HONG, Ming YANG, Shaoping LI, Jing ZHAO

**Affiliations:** 1.澳门大学中药质量研究国家重点实验室, 澳门 999078; 1. State Key Laboratory for Quality Research of Chinese Medicine, University of Macau, Macao 999078, China; 2.江西国药有限责任公司, 江西 南昌 330096; 2. Jiangxi Guoyao Pharmaceutical Co., Ltd., Nanchang 330096, China

**Keywords:** 指纹图谱, 质控指标, 主成分分析, 聚类分析, 发酵虫草菌粉, fingerprints, quality control index, principal component analysis (PCA), hierarchical clustering analysis, fermented *Cordyceps* powder

## Abstract

《中国药典》收载的发酵虫草菌粉产品的质量标准中,规定以鸟苷、腺苷、尿苷的含量作为评价相关产品质量的标准。但除此之外,还有许多其他的核苷类成分对发酵虫草菌粉质量控制的影响尚未被探讨。为探究发酵虫草菌粉及产品质控指标选择的合理性,采用超高效液相色谱-紫外检测法对19批发酵虫草菌粉及产品中9种核苷成分(尿嘧啶、胞苷、鸟嘌呤、尿苷、腺嘌呤、肌苷、鸟苷、胸苷、腺苷)进行了定量分析,建立了发酵虫草菌粉样品的指纹图谱,并结合统计学提供了一种分析指标性成分的方法。通过优化样品的提取方法,选择超声提取法制备19批发酵虫草菌粉及产品的供试液;采用Agilent Eclipse Plus C18色谱柱(150 mm×4.6 mm, 3.5 μm)进行色谱分析,以甲醇-水为流动相梯度洗脱,对方法的校正曲线、准确度、精密度、重复性和回收率进行了验证。结合对照品指认了指纹图谱中的9个核苷峰,并采用外标一点法测得了各核苷成分的含量。使用化学模式识别对指纹图谱中的共有峰进行分析,聚类分析和主成分分析得到了同样的分类结果:19批样品共分为5类,其中同一发酵虫草菌粉因工艺差异可分为2类,而心肝宝胶囊、百令胶囊、宁心宝胶囊则各单独分为1类。同时,使用主成分分析获得了各样品中的指标性成分,分别为尿苷、鸟苷、腺苷、腺嘌呤、尿嘧啶,并使用聚类分析再次进行确证,验证了指标性成分的合理性。

## Abstract (en)

Currently, guanosine, adenosine, and uridine contents are specified as the quality criteria for related products in the quality standards for fermented *Cordyceps* powder preparations included in the 2020 edition of Chinese Pharmacopoeia. However, there are many other nucleosides in fermented *Cordyceps* powder, whose effect on the quality control has not yet been discussed. In this study, an ultra-performance liquid chromatography-ultraviolet detection (UPLC-UV) method was used for the quantitative analysis of 9 nucleosides (uracil, cytidine, guanidine, uridine, adenine, inosine, guanosine, thymidine, and adenosine) in 19 batches of fermented *Cordyceps* powder samples and products, and the corresponding fingerprints were established. In addition, a method for analyzing the index components was proposed based on statistics. By optimizing the sample extraction method, ultrasound-assisted extraction was selected to process 19 batches of samples. Chromatographic analysis was performed on an Agilent Eclipse Plus C18 column (150 mm×4.6 mm, 3.5 μm) using methanol and water as the mobile phases under gradient elution. The method was validated based on the calibration curves, accuracy, precision, repeatability, and recovery. The fingerprints of the 19 batches of samples were established, and 16 common peaks were obtained. Among them, nine nucleoside peaks were identified by standards, and their concentrations were determined by the external standard one-point method. Similarity evaluation of the fingerprints was conducted; the similarities of the 19 batches of samples were greater than 0.9. Then, chemical pattern recognition was performed. The same classification results were obtained by hierarchical clustering analysis (HCA) and principal component analysis (PCA). Thus, the samples could be segregated into five classes, and the fermented *Cordyceps* powders were classified as two types with different fermentation processes. Xinganbao capsules, Bailing capsules and Ningxinbao capsules were each separately classified into one class. This indicated that the chemical recognition pattern could effectively distinguish between the fermented *Cordyceps* powder and different products. PCA was used to calculate the weight value of each common peak for the first time, and the index components among the samples were selected according to the weight value. Finally, the selected index components were used to re-cluster the samples. The results were consistent with those obtained on the basis of the 16 common peaks, thus verifying the rationality of the index components. Therefore, uridine, guanosine, adenosine, adenine, and uracil are recommended for use as evaluation indicators for fermented *Cordyceps* powder and products, allowing for better distinction between the products on the market. In summary, the combination of liquid chromatographic fingerprints and chemical pattern recognition can provide a simple and reliable method for the analysis and quality control of fermented *Cordyceps* powder and products.

发酵虫草菌粉(fermented *Cordyceps* powder)是从天然冬虫夏草中分离出的虫草菌经液体发酵培养所得菌丝体的干燥粉末^[1]^。发酵虫草菌粉的主要化学成分与冬虫夏草类似,但价格相对较低,常作为天然虫草的替代品^[2,3,4]^,具有补益肺肾、秘精益气的功效^[5]^。市面上以发酵虫草菌粉为原料的制剂有金水宝胶囊、百令胶囊、宁心宝胶囊、心肝宝胶囊等^[6]^。核苷类成分是发酵虫草菌粉中的关键成分,2020版《中国药典》中收载的发酵虫草菌粉制剂金水宝片/胶囊的质量标准中规定以鸟苷、腺苷、尿苷的总含量作为评价相关产品质量的标准,百令胶囊以腺苷含量作为测定标准^[1]^。但发酵虫草菌粉中除上述3种核苷类成分外,还有许多其他的核苷类成分^[7]^,这些成分对于菌粉和相关产品质量评价的影响尚未被讨论。因此,本文希望通过对发酵虫草菌粉及产品中核苷类成分的分析,探讨其质控指标选择的合理性。

中药指纹图谱能充分反映中药的化学成分信息,具有整体性和全面性,是评价中药质量的常用方法^[8,9,10,11]^。统计学方法可以对指纹图谱信息进行处理,结合多个指标对中药质量进行分类和综合评价,其中常用的有聚类分析和主成分分析^[12,13,14]^。在对指纹图谱的分析中,本文首次在主成分分析中引入权重计算来获得发酵虫草菌粉共有峰的权重值,以权重值的大小选择其中的指标性成分。在得到指标性成分后,使用聚类分析进一步验证指标性成分能否代替共有峰实现对不同的样品的区分,从而判断指标性成分的选择是否可信。

本文采用超高效液相色谱-紫外检测法建立了发酵虫草菌粉和市面上3种胶囊产品的指纹图谱,测定了其中9种核苷类成分的含量。通过化学模式识别对指纹图谱进行评价,并结合统计学提供了一种新的分析、验证指标性成分的方法,为发酵虫草菌粉和产品的质控指标选择提供了科学依据。

## 1 实验部分

### 1.1 仪器、试剂与材料

Ultimate 3000超高效液相色谱系统,包括自动进样器、在线脱气机、四元梯度泵、柱温箱和UV检测器(Thermo,美国);加压溶剂萃取仪(Dionex,美国);超声清洗仪(Branson,美国);十万分之一分析天平(Mettler Toledo,瑞士); Milli-Q Direct一体化超纯水机(Millipore,德国)。

对照品尿嘧啶(uracil)、胞苷(cytidine)、鸟嘌呤(guanine)、胸苷(thymidine)、尿苷(uridine)、腺嘌呤(adenine)、肌苷(inosine)(纯度≥99%, Sigma-Aldrich,美国),鸟苷(guanosine)、腺苷(adenosine)(纯度≥98%, 北京索莱宝有限科技公司),甲醇(色谱纯,Merck,德国)。

发酵虫草菌粉原料10批(依次标记为S1~S10),心肝宝胶囊3批(批号:18190401、18200402、18200401,依次标记为S11~S13,河北长天药业有限公司),百令胶囊3批(批号:1911366、1908318、1907333,依次标记为S14~S16,杭州中美华东制药有限公司),宁心宝胶囊3批(批号:200401、200402、200103,依次标记为S17~S19,上海普康药业有限公司),每个胶囊产品取20粒,分别混合均匀后作为待测样品。

### 1.2 实验方法

1.2.1 色谱条件

采用Agilent Eclipse Plus C18分析柱(150 mm×4.6 mm, 3.5 μm),柱温25 ℃,流速1.0 mL/min,流动相甲醇(A)和水(B)。梯度洗脱程序:0~8.0 min, 0%A; 8.0~8.5 min, 0%A~5%A; 8.5~20.0 min, 5%A~25%A; 20.0~22.0 min; 25%A~100%A。进样量5 μL,检测波长260 nm。

1.2.2 对照品溶液制备

分别称取对照品适量,精密称定,加水溶解制成质量浓度为10.7 mg/L尿嘧啶、3.8 mg/L胞苷、10.4 mg/L鸟嘌呤、51.8 mg/L尿苷、10.1 mg/L腺嘌呤、2.1 mg/L肌苷、40.6 mg/L鸟苷、3.7 mg/L胸苷、50.5 mg/L腺苷的对照品溶液。

1.2.3 供试品溶液制备

取样品粉末0.5 g,精密称定。加入水25 mL,称量。超声(功率881 W,频率43 kHz)处理10 min,放冷,加水补足减少的质量,以3000 r/min离心15 min,取上清液过0.45 μm滤膜待用。

## 2 结果与讨论

### 2.1 样品提取方法的考察

分别采用超声辅助提取法和加压溶剂萃取法提取发酵虫草菌粉样品,在优化条件下,两种提取方法得到的9种核苷含量无显著差异(见图1a)。由于超声辅助提取样品的操作更为简单,且超声提取仪器更常见^[15]^,因此选择超声辅助提取作为样品的提取方法。同时实验对超声提取时间进行了考察(10、20、30、40 min),结果显示,不同超声提取时间得到的核苷含量无显著差异(见图1b)。因此选择室温下超声辅助提取10 min作为样品的提取条件,并以此条件提取全部样品。

**图1:**
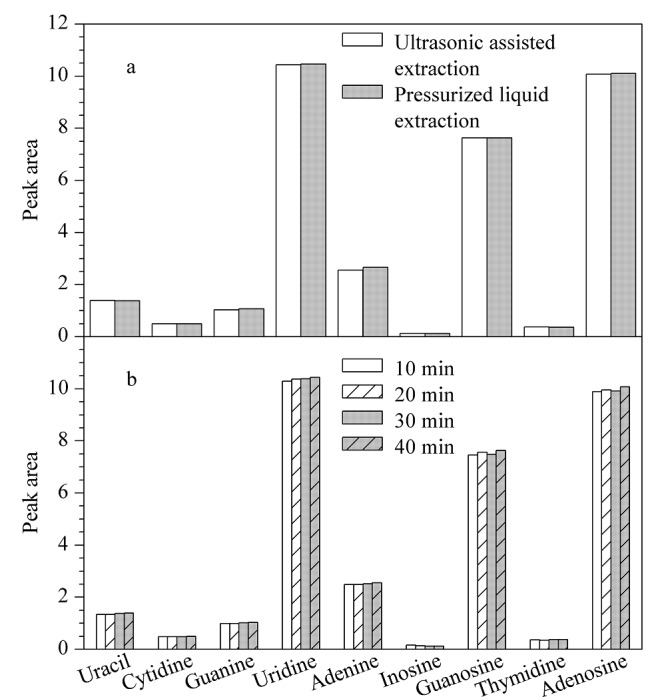
不同(a)提取方法和(b)超声提取时间对样品中 核苷峰面积的影响

### 2.2 方法学考察

分别精密吸取适量对照品溶液,加水稀释成5个不同浓度的对照品溶液,按1.2.1节方法进样测定,以峰面积(*Y*)对对照品质量浓度(*X*, mg/L)进行线性回归。9个组分在各自的范围内线性关系良好,回归系数(*R*^2^)均不小于0.999(见表1)。

**表1:** 9个核苷成分的回归方程、线性范围、相关系数

Nucleoside	Regression equation	Linear range/ (mg/L)	R^2^
Uracil	Y=0.2472X+0.0075	2.1-32.1	1.0000
Cytidine	Y=0.1041X+0.0010	0.8-11.5	1.0000
Guanine	Y=0.1881X-0.0484	2.1-31.2	0.9999
Uridine	Y=0.1383X+0.0337	10.4-155.4	1.0000
Adenine	Y=0.3052X+0.0644	2.0-30.4	0.9998
Inosine	Y=0.1071X+0.0002	0.4-6.3	1.0000
Guanosine	Y=0.1337X+0.0287	8.1-121.9	0.9997
Thymidine	Y=0.1324X+0.0012	0.7-11.0	0.9999
Adenosine	Y=0.1842X+0.0525	10.1-151.5	1.0000

*Y*: peak area; *X*: mass concentration, mg/L.

取混合对照品溶液连续进样6次,测定检测系统精密度;取发酵虫草菌粉1号样品,按1.2.3节方法平行制备供试品溶液6份,进样测定重复性;分别在供试品溶液制备后0、4、8、12和24 h进样,测定样品稳定性;精密度试验、重复性试验、稳定性试验中9个组分相对保留时间的RSD均小于1.0%,相对峰面积的RSD均小于3.0%,表明仪器的精密度、方法的重复性、样品的稳定性良好。

取发酵虫草菌粉样品6份,分别精密加入与样品等量的肌苷、胞苷、胸苷、尿嘧啶、鸟嘌呤、腺嘌呤、鸟苷、尿苷、腺苷对照品,按优化的超声提取法处理,进样测定,计算回收率。各核苷组分的平均回收率为95.6%~101.2%,表明方法的准确度良好。

### 2.3 含量测定

将19批发酵虫草菌粉及产品分别按1.2.3节方法制备供试品溶液,按1.2.1节的色谱条件分别进样测定,其中9种混合标准品溶液的色谱图见图2a, 1号样品的色谱图见图2b。依据保留时间鉴定样品中各核苷成分,并记录260 nm波长下各成分的色谱峰面积。按外标一点法分别计算各组分的含量,结果见附表1(详见http://www.chrom-China.com)。不同产品中各核苷成分的含量区别较大,但尿苷、鸟苷、腺苷的含量均比较高。

**图2:**
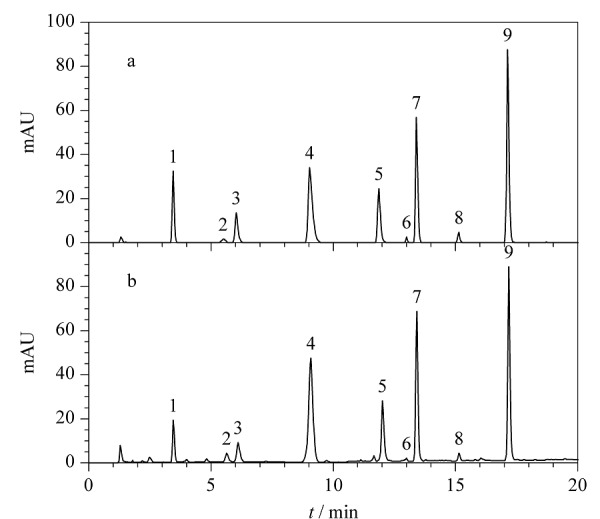
(a)混合对照品溶液及(b)1号发酵虫草菌粉 样品的指纹图谱

### 2.4 指纹图谱的建立

将19批发酵虫草菌粉及产品的色谱图导入至“中药色谱指纹图谱相似度评价系统(2012.1版本)”软件进行处理,生成对照指纹图谱(R)和样品的指纹图谱(见图3)。19批发酵虫草菌粉样品的色谱图中共识别出16个主要的共有特征峰,根据对照品将9个共有峰分别鉴定为尿嘧啶、鸟嘌呤、尿苷、胸腺嘧啶、腺嘌呤、肌苷、鸟苷、胸苷、腺苷,样品的各共有峰相对保留时间RSD均小于1.0%,共有指纹峰的峰面积百分比大于99%。

**图3:**
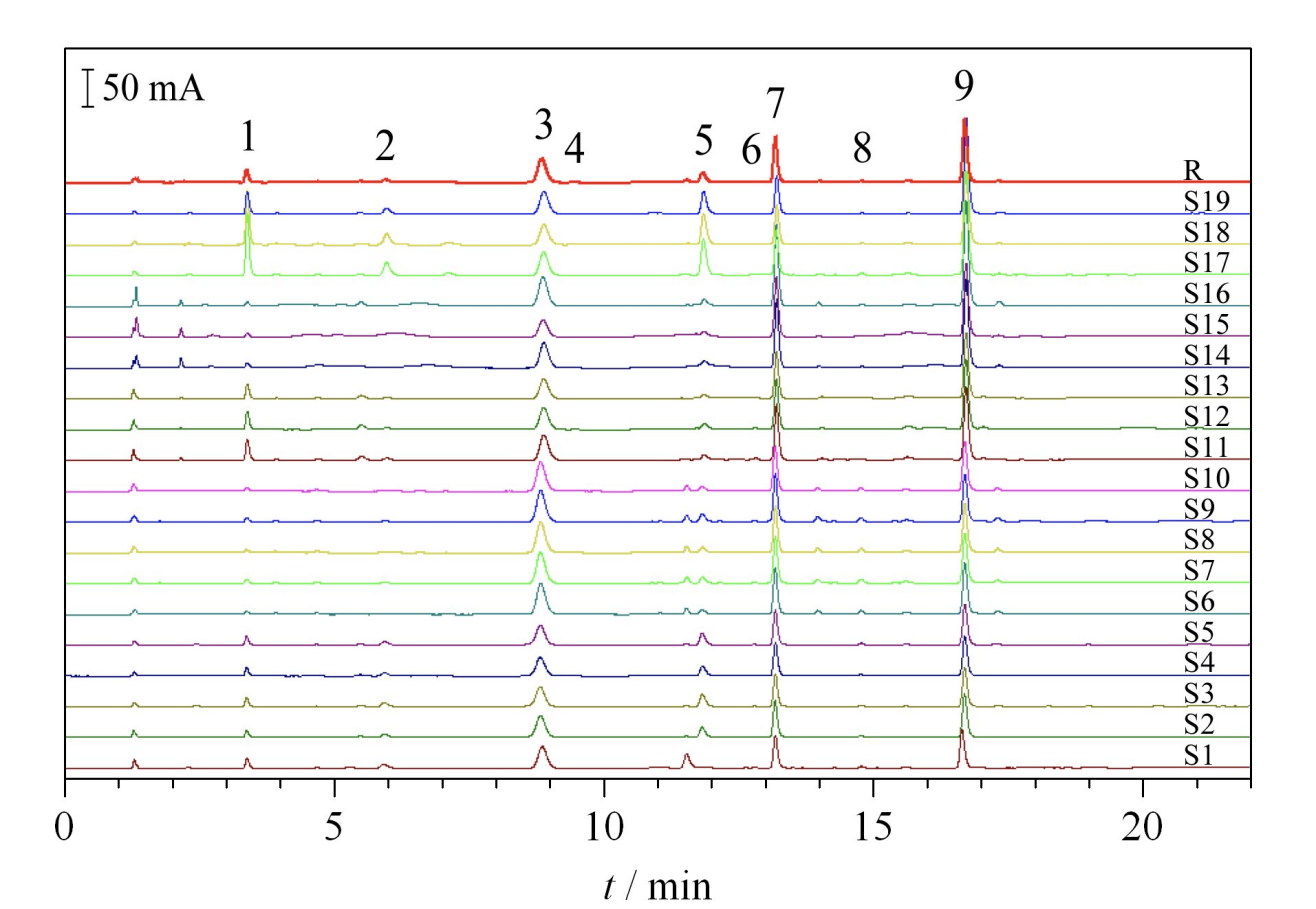
发酵虫草菌粉样品及产品的指纹图谱

### 2.5 指纹图谱评价

2.5.1 指纹图谱的相似度评价

采用“中药色谱指纹图谱相似度评价系统(2012.1版本)”软件,将对照图谱作为参照图谱,计算19批发酵虫草菌粉及产品指纹图谱的相似度。各共有峰相对保留时间RSD均小于1.0%, 19批样品的相似度均大于0.9,相似度结果见表2。要进一步分析其中的代表性成分,须进行化学模式识别。

**表2:** 19批发酵虫草菌粉样品及产品的相似度

No.	Similarity	No.	Similarity	No.	Similarity
S1	0.986	S8	0.979	S15	0.956
S2	0.993	S9	0.977	S16	0.974
S3	0.988	S10	0.983	S17	0.903
S4	0.995	S11	0.988	S18	0.903
S5	0.991	S12	0.985	S19	0.948
S6	0.982	S13	0.982		
S7	0.982	S14	0.974		

2.5.2 主成分分析

采用MATLAB软件,将共有峰的峰面积数据作为变量,分别对发酵虫草菌粉和3种产品各自进行主成分分析,并计算每个成分的权重值。权重值代表该成分峰对主成分的贡献,权重值越高,对主成分贡献越大,将权重值大于0.1的成分作为样品的指标性成分。经分析,各样品中指标性成分如下:发酵虫草菌粉原料中的尿苷(0.24)、鸟苷(0.14)、腺苷(0.14)、尿嘧啶(0.10);心肝宝胶囊样品中的腺苷(0.20)、尿苷(0.18)、鸟苷(0.17)、尿嘧啶(0.12); 百令胶囊样品中的尿嘧啶(0.19)、鸟苷(0.18)、腺嘌呤(0.16)、腺苷(0.14)、尿苷(0.10);宁心宝胶囊样品中的尿嘧啶(0.22)、腺苷(0.21)、腺嘌呤(0.20)、尿苷(0.10)、鸟苷(0.10)。

对19个样品同时进行主成分分析,其主成分分析得分图显示,19个样品可分为5类(见图4),分别为:S1~S5、S6~S10、S11~S13、S14~S16和S17~S19。其中S1~S10为10批发酵虫草菌粉样品,由两种发酵工艺制得,不同工艺得到的产品质量存在差异,因此聚为两类;S11~S13、S14~S16和S17~S19分别为心肝宝胶囊、百令胶囊、宁心宝胶囊3种产品,来自不同厂家,其生产工艺和生产环境不同,均会造成产品之间的差异。

**图4:**
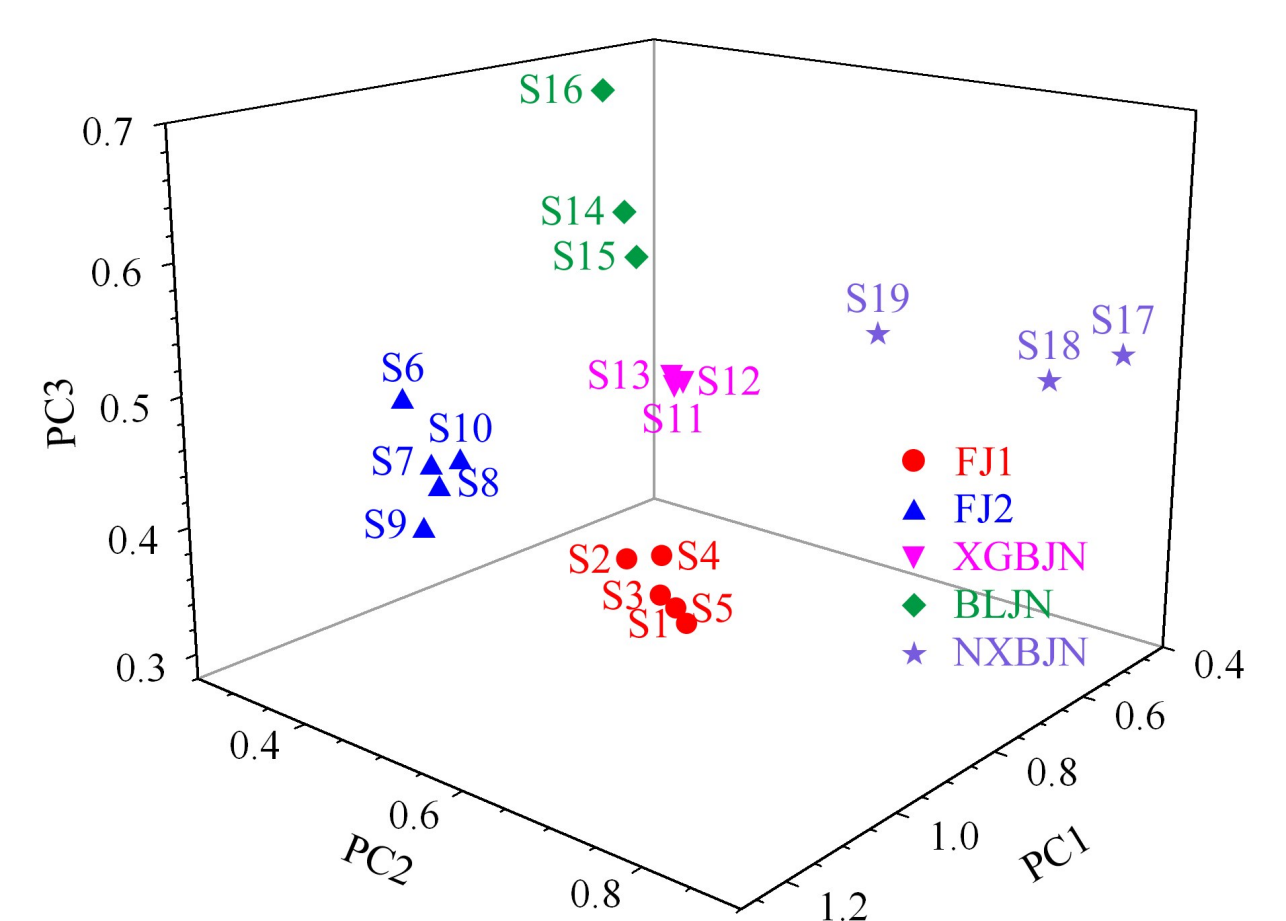
发酵虫草菌粉样品及产品的主成分分析得分图

而同种产品不同批次之间的一致性较好,因此各产品单独聚为一类。对19批样品同时进行权重计算,有5个成分权重值大于0.1,分别为:尿苷(0.28)、鸟苷(0.14)、尿嘧啶(0.13)、腺苷(0.10)、腺嘌呤(0.10),即这些成分可以作为区别全部样品的指标成分。

2.5.3 聚类分析

将19批样品的共有峰峰面积作为变量,导入SPSS软件中进行聚类分析,结果见图5a。由结果可知,19批样品可聚为5类,与主成分分析结果一致。同时,使用主成分分析中筛选出的5个指标成分对19批样品进行聚类分析,得到的结果如图5b,与使用全部共有峰进行聚类分析的结果一致,说明仅使用这5种指标成分就可以实现对不同的发酵虫草菌粉和产品的区分。

**图5:**
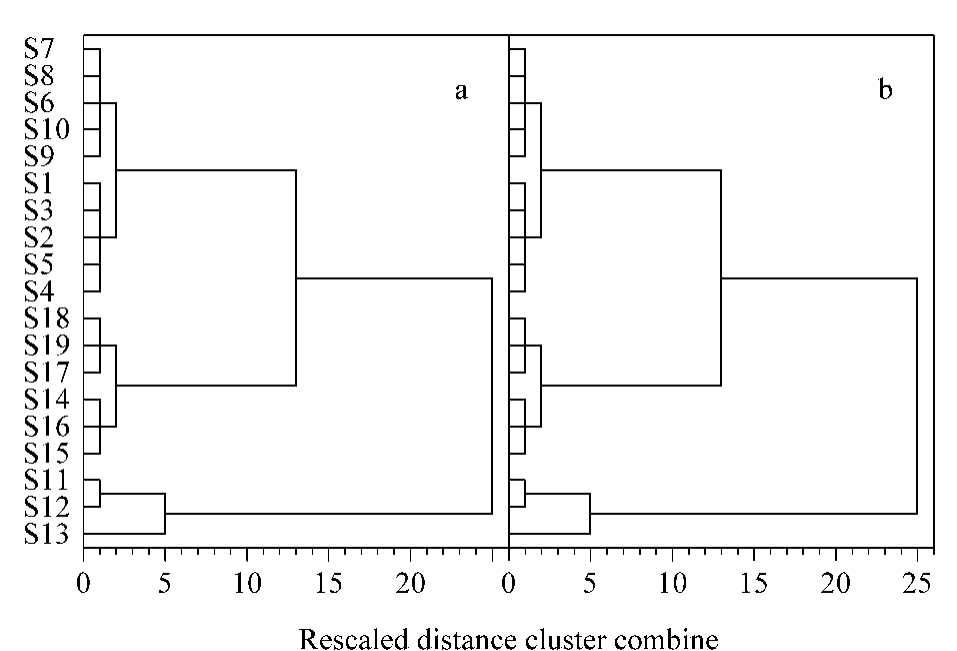
发酵虫草菌粉样品及产品的(a)共有峰及(b)指标 成分的聚类分析树状图

## 3 结论

本文建立了一种发酵虫草菌粉原料和市场上3种主要产品的指纹图谱,得到了16个共有峰,测定了其中9个核苷的含量,并结合统计学方法分析得到了指标性成分,分别为尿苷、鸟苷、腺苷、腺嘌呤、尿嘧啶。以上结果表明,该方法可以定量分析发酵虫草菌粉中多种核苷成分。同时,利用液相色谱指纹图谱结合化学模式识别,可以分析与评价发酵虫草菌粉及产品中的指标性成分,为其质量控制提供简单、可靠的方法。将该分析方法进一步扩展,可以为其他中药指纹图谱的建立、指标成分的分析提供参考,为中药质量评价提供实验依据。
